# The Journal Club

**DOI:** 10.4103/0253-7613.64494

**Published:** 2010-04

**Authors:** R K Dikshit

**Affiliations:** Compilation

What happens to theses submitted as a part of post-graduate medical examination? A study conducted retrospectively for last five years at a University Medical College in India has observed that only 30% of theses are published in indexed, peer reviewed journals (*Dhaliwal U, Singh N, Bhatia A. Masters theses from a university medical college: Publication in indexed scientific journals. Indian J Ophthalmol 2010; 58: 101-4*). This too may happen after 8 to 74 months of their submission. Interestingly, the authors have observed that the post-graduate student is the first author in papers from only half of these theses! The authors feel that a low publication rate, apart from other things, represents a waste of manpower, money and other resources.

Rapid urease test (RUT) is widely used for diagnosis of *H. pylori* infection. It gives quick results with an adequate sensitivity and specificity. However, several groups of drugs (e.g. proton pump inhibitors, antibiotics, H2 receptor antagonists and bismuth) may influence the test results. Whether results can be altered by non-steroidal anti-inflammatory drugs (NSAIDs) has been investigated in a case control study (*Foroutan M, Loloei B, Irvani S, Azargashb E. Accuracy of rapid urease test in diagnosing Helicobacter pylori infection in patients using NSAIDs. Saudi J Gastroenterol 2010;16:110-12*). It included 210 patients divided in three groups (1-negative RUT and a history of NSAID consumption, 2-negative RUT and no history of NSAID consumption and 3-positive RUT with (32 patients) or without (38 patients) NSAIDs consumption). The groups matched demographically. NSAIDs used were acetyl salicylic acid, diclofenac, ibuprofen, piroxicam, indomethacin and naproxen, consumed for a varying period of one to four weeks. The overall performance of RUT was nearly same in all the groups. Authors have concluded that the results of RUT are not affected by NSAID use. They have, however, recommended that a similar study should be conducted in larger number of patients.

Ginger (*Zingiber officinale*) causing subacute thyroditis! In an interesting publication (*Sanavi S, Afshar R. Subacute thyroiditis following ginger (Zingiber officinale) consumption. Int J Ayurveda Res 2010;1:47-48*) the authors describe the case of a 34 year old female who developed the features of thyrotoxicosis (severe pain at the back of the mouth and neck radiating to jaw, difficulty in swallowing, hoarse voice, mild fever, palpitation, diffusely enlarged and tender thyroid gland) twice within a period of 18 months. Thyroid function tests revealed thyrotoxicosis on both the occasions and a diagnosis of subacute thyroiditis was made. During first episode she gave the history of ginger powder consumption (1 tsf) with honey for 10 nights and on second occasion she developed the symptoms after taking a ginger candy (30 g). After first episode she became normal within five months (treated with NSAIDs, propranolol and prednisolone) and second time within four weeks (propranolol only). Authors speculate if an inhibitory effect of ginger on metabolic rate and adenylate energy status may damage the integrity of membranes surrounding the thyroid hormone in follicles and eventually release the hormone into circulation. Alternatively, it may also be an autoimmune or allergic reaction.

Why do we not do enough of medical research in India? In an editorial (*Dalvi B. Are we shy of clinical research in India? Ann Pediatr Card 2009;2:109-110*), the author traces the cause right from our formative years in family and schools ('instinct to explore nipped in the bud' , 'intellectually unchallenging curriculum and examination pattern') to the medical colleges ('discouraging academic inquiry'), medical practice ('overburdened clinical responsibilities') and a variety of other factors ('lack of funds, poorly paid research scientists and a general disinterest of the society and media in medical research'). The author feels that although the currently booming 'clinical research' may not be the 'real' one, it may still provide us the necessary orientation and discipline. Research is, however, important to survive in this competitive world. It is recommended that for this purpose the medical fraternity should work together (in cooperative groups) pooling the available resources and in an integrative partnership with the private philanthropies, government, private health care sector and the industry.

Those working at pesticide retail shops are more likely to have a significant slowing of motor nerve conduction velocity as compared to others. In a small but interesting study (*Kesavachandran C, Pathak MK, Fareed M, Bihari V, Mathur N, Srivastava AK. Health risks of employees working in pesticide retail shops: An exploratory study. Indian J Occup Environ Med 2009;13:121-6*)conducted on 20 subjects working at equal number of randomly selected pesticide retail shops from two districts matched with 18 controls, it was revealed that the exposed persons also suffered from a significant impairment of respiratory and gastrointestinal functions. Ocular, cardiovascular and musculo-skeletal abnormalities were also noted among the study subjects. The shops mainly stocked organophosphates, organochlorines, carbamates and pyrethroids. Authors found that the protective devices as well as other protection practices were not employed despite the fact that all the shopkeepers were aware of pesticide toxicity! Authors recommend a better safety culture at our work places.

A dermatologic safety profile of seventeen commonly used detergents found in Indian market (laundry-12 and dish wash-5) has been evaluated in 30 volunteers (*Austoria AJ, Lakshmi C, Srinivas CR, Anand CV, Mathew AC. Irritancy potential of 17 detergents used commonly by the Indian household. Indian J Dermatol Venereol Leprol 2010;76:249-53*). The authors have used a 24-hour patch testing for this purpose and they feel that this may be ideal as a screening test to help patients avoid the detergents that result in an irritant response. Most detergents are non-soap anionic surfactants and they may disrupt the stratum corneum barrier function to produce dermatitis. Inherent irritancy of the product, its pH and perhaps some other factors decide the irritancy potential of a detergent. In this study, the authors found that significant differences existed among various detergents as far as erythema, dryness and wrinkling is concerned but not for the edema. Many of the well known, popular, national brands scored better than others!

